# Reaction of O_2_ with α‑Aminoalkyl Radicals Derived from Tetrahydropyridines

**DOI:** 10.1021/acs.jpca.5c02967

**Published:** 2025-06-11

**Authors:** Paul Venturo, Neal Castagnoli, James M. Tanko

**Affiliations:** Department of Chemistry, 1757 Virginia Polytechnic Institute and State University, Blacksburg, Virginia 24061, United States

## Abstract

Tetrahydropyridines, such as the Parkinsonian-symptom-inducing neurotoxin 1-methyl-4-phenyl-1,2,3,6-tetrahydropyridine (MPTP) and its derivatives are unique in that they are the only known tertiary amines that are monoamine oxidase (MAO) substrates or inhibitors. These compounds all possess an exceptionally weak C–H bond at C-6, which is alpha both to a nitrogen lone pair and a C=C. Radicals (R^•^) derived from hydrogen abstraction at this position are exceptionally stable. Similar to other α-aminoalkyl radicals, these radicals react with oxygen at a nearly diffusion-controlled rate and are readily oxidized. Evidence suggests that while the initial product of this reaction is likely a peroxyl radical resulting from radical trapping (ROO^•^), a dihydropyridinium species (DHP^+^) is produced through what can be described as an overall inner-sphere electron transfer process. These results imply another role for O_2_ in the MAO catalytic cycle. In addition to regenerating the flavin moiety in MAO, O_2_ may also be directly involved in oxidizing the substrate radical.

## Graphical abstract

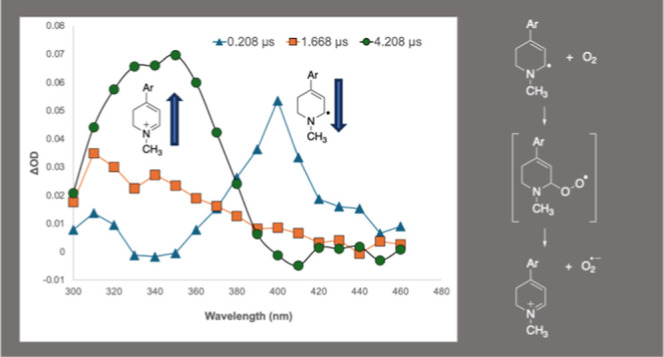

## Introduction

Monoamine oxidase A (MAO-A) and -B (MAO-B) catalyze the oxidation of various neurotransmitters including dopamine, norepinephrine, epinephrine, and serotonin. The overall reaction is a two-electron α-carbon oxidation, H_2_N–CH_2_R → HN=CHR, coupled to the two-electron reduction of the flavin cofactor FAD to FADH_2_ to complete the catalytic cycle.^1,2^ Several mechanisms have been proposed to account for the initial stages of the mechanism of MAO-catalyzed oxidations including (a) nucleophilic,^3^ (b) hydride transfer,^4,5^ and (c) single electron transfer (SET).^6–10^ Generally, the polar mechanisms (nucleophilic or hydride transfer) seem to have become favored over SET. While there are a few recent reports advocating for SET,^11–13^ most recent studies and reviews have favored almost exclusively the polar pathways (nucleophilic and or hydride transfer mechanisms).^2,14–16^

However, there is an important issue that has received scant attention—the reaction of MAO with tertiary amines, particularly tetrahydropyridines. Generally, tertiary amines are not MAO substrates; steric crowding prevents reaction from occurring via the “accepted” polar mechanisms. Yet, the potent neurotoxin 1-methyl-4-phenyl-1,2,3,6-tetrahydropyridine (MPTP) and other tetrahydropyridines are the only known tertiary amines with good MAO substrate (or inhibitor) properties.^17,18^ Tertiary amines such as deprenyl (selegiline), clorgyline, and pargyline are also known MAO inhibitors possibly because they possess analogous molecular functionality.^19,20^

Using the biomimetics 5-ethyl-3-methyllumiflavinium perchlorate^21^ and 3-methyllumiflavin^22^ to mimic the flavin active site in MAO, we recently published compelling evidence in support of an SET mechanism and developed a new hypothesis (Figure 1) to explain why for certain substrates, such as MPTP, an electron transfer process becomes viable. At the core of this hypothesis is the belief that the α-C=C in MPTP (and derivatives) dramatically lowers the p*K*_a_ of the corresponding aminyl radical cations, from ca. 8 for a “normal” tertiary amine radical cation to as low as −5 for MPTP^•+^. Thus, although the electron transfer process is a thermodynamically unfavorable equilibrium, it is driven toward products in the context of LeChatlier’s principle because of an extremely favorable deprotonation step.

**Figure 1:**
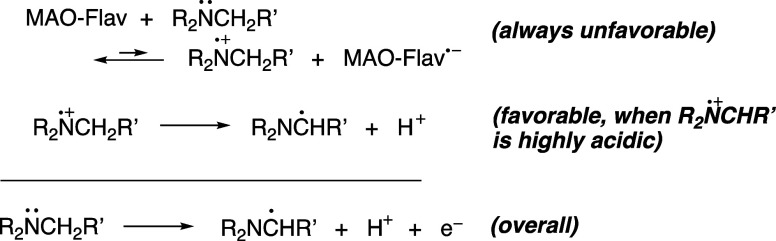
Single electron transfer (SET) hypothesis for the MAO-catalyzed oxidation of tetrahydropyridines; an unfavorable electron transfer is driven by an extremely favorable deprotonation of a highly acidic radical cation.

The traditional role of O_2_ in the mechanism of MAO catalysis involves reoxidation of the flavin cofactor to complete the catalytic cycle. In earlier work, we suggested (Figure 2) another possible role for O_2_ in the SET mechanism, oxidation of MPTP^•^ (the α-amino alkyl radical purportedly produced by proton coupled electron transfer) to form a dihydropyridinium species.^21,22^ As noted, MPTP is a potent neurotoxin, and MPDP^+^ is the precursor to the pyridinium ion (MPP^+^, formed via autoxidation) which leads to nerve damage in humans and animals. While the literature is clear that the reaction of O_2_ with α-aminoalkyl radicals occurs at a nearly diffusion controlled rate, the reaction is generally assumed to be radical trapping to yield a peroxyl radical (R^•^ + O_2_ → RO_2_^•^), as opposed to single electron transfer.^23^

**Figure 2:**
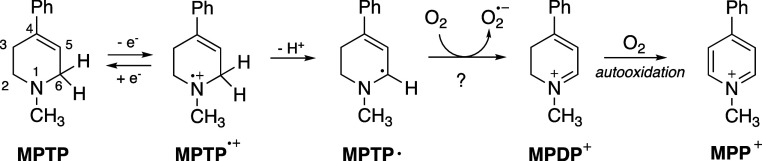
Proposed mechanism for the MAO-catalyzed oxidation of MPTP (and derivatives).

The objective of this work is to directly determine whether MPTP^•^ and related radicals react with O_2_ to produce a dihydropyridinium species. Through deuterium labeling studies and nanosecond laser flash photolysis (LFP), it was previously shown that hydrogen atom abstraction from MPTP by the *t*-butoxyl radical ((CH_3_)_3_CO^•^) occurs almost exclusively at C-6 (by far, the weakest C–H bond in the molecule),^24^ and that the resulting radical (MPTP^•^) has a characteristic absorption at 385 nm that can be used to follow the kinetics of the reaction.^25,26^ Accordingly, in this report we utilized laser flash photolysis to generate ^*t*^BuO^•^ (from the corresponding peroxide) to generate aminoxyl radicals from various tetrahydropyridines related to MPTP, allowing their rate constants for reaction with O_2_ to be determined. Moreover, because both the aminoxyl radicals and dihydropyridinium species resulting from electron transfer often have unique UV signals, it was possible to assess whether the reaction with O_2_ involved electron transfer. (In the case of MPDP^+^, λ_max_ = 345 nm).^27^ Molecular orbital calculations were also performed to determine λ_max_ for both the MPTP-derived radicals and dihydropyridinium species, and the corresponding oscillator strengths (*f*) as predictor of the extinction coefficient of each entity.

## Methods

### Materials

All chemicals and solvents used were at the highest purity available. All MPTP derivatives were synthesized according to literature procedures and stored as their respective oxalate or hydrochloride salts: **1**(**a**–**c**),^28^ **2**, **3**, **5**,^9^ and **4**.^29^ The salts were treated using aqueous potassium carbonate solutions and the generated free amine was extracted using methylene chloride prior to the production of the LFP solutions. Caution: MPTP is a known nigrostriatal neurotoxin and should be handled with care under a ventilated hood and with the proper personal protective equipment. Procedures for the safe handling of MPTP have been documented. Although the compounds chosen for study have no known human toxicity, these precautions were followed nonetheless.

### Instrumentation

All LFP experiments were performed using an Applied Photophysics LKS.80 spectrometer using the third harmonic (355 nm) of a Continuum Surelite I-10 Nd:YAG laser with a pulse duration between 4 and 6 ns. Transient signals were monitored by an HP Infinium digital oscilloscope and analyzed with the Applied Photophysics Kinetic Workbench software package (v 3.0.1).

To determine the rate constant for hydrogen abstraction (*k*_H_) from each tetrahydropyridine and the transient UV/vis spectrum of the resulting radical, a deoxygenated solution of acetonitrile with 7.5% di-*tert*-butyl peroxide with varying concentrations of substrate was prepared, following the procedure outlined in a previous paper by Suleman et al.^25^ The samples were deoxygenated by bubbling argon gas through the solution for 15 min.

Transient absorption spectra for the tetrahydropyridine-derived radicals were constructed using LFP with deoxygenated samples containing approximately 10 mM of the tetrahydropyridine. Each sample excitation was followed by a 30 s resting period between readings. The transient spectra were constructed using the ΔOD at times between 800 and 1000 ns. The rate constant for hydrogen abstraction (*k*_H_) was determined using 3–5 different amine concentrations monitoring λ_max_ of the produced radical determined by a transient absorption using a 10 mM sample prior.

To characterize the reaction between the MPTP-derived radicals and O_2_, two samples containing 7.5 mM of an MPTP derivative in acetonitrile containing 7.5% di-*tert*-butyl peroxide were utilized. One sample was deoxygenated as described above, while the other was air-saturated. The transient absorption spectra of these two samples were compared to observe whether the emergence of a new λ_max_ attributable to formation of a dihydropyridinium species would occur in the oxygenated sample at a longer time scale. To determine the rate constant for the reaction with O_2_ (*k*_O_2__), a procedure used by Lalevée et al.^23^ was implemented in which the observed rate of two identical samples is taken, with one under aerobic conditions and another deoxygenated with inert gas. As the concentration of oxygen in the deoxygenated sample is zero, and the concentration of oxygen in air-saturated acetonitrile is known (1.91 mM in CH_3_CN),^30^ *k*_O_2__ is readily determined.

### Molecular Orbital Calculations

Computational calculations of absorption of R^•^ and DHP^+^ were performed using the Gaussian 09 software.^31^ The geometry was optimized by density functional theory (DFT) at the B3LYP level of theory with the 6-31G* basis set. The optimized structures were used for the calculation of the absorption spectra by time-dependent functional theory (TDDFT) using the B3LYP level of theory and the 6-31G* basis set with the addition of a conductor-like polarizable continuum model (CPCM) for acetonitrile.

## Results and Discussion

In place of MPTP, which is a known nigrostriatal neurotoxin, tetrahydropyridine derivatives **1–5** (Figure 3) were chosen for study because these compounds have no known human toxicity. Each of these derivatives possess the same structural features (e.g., a CH_2_ that is both allylic and α- to nitrogen, highlighted in blue) which are critical to MPTP reactivity.

**Figure 3:**
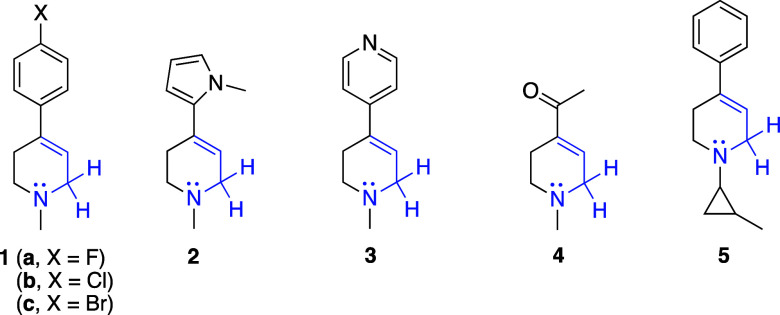
Tetrahydropyridines used in this study. The features common to MPTP which are critical to MPTP reactivity are highlighted in blue.

*t*-Butoxyl radical (^*t*^BuO^•^) was generated from di-*t*-butylperoxide using the LFP protocol developed by Scaiano et al. (Figure 4).^32,33^ This involves a short laser pulse (4–6 ns at 355 nm) directed at a sample containing the peroxide (7.5%) and tetrahydropyridine derivative in acetonitrile. Photolysis produces ^*t*^BuO^•^, which decays via β-scission ((CH_3_)_3_CO^•^ → (CH_3_)_2_C=O + CH_3_^•^), hydrogen abstraction from solvent (minor pathway), and for the purposes of this study, hydrogen abstraction from the MPTP derivative. For MPTP, it has been shown previously that the major reaction (>70%) between ^*t*^BuO^•^ and MPTP involves hydrogen atom abstraction from C-6, i.e., the aforementioned reactive CH_2_.^25^ Most conveniently, the resulting MPTP-derived radical exhibits a characteristic absorption at 385 nm that can be used to monitor the kinetics. Under these conditions, the observed (pseudo) first order rate constant is *k*_obs_ = *k*_o_ + *k*_H_[amine], where *k*_o_ accounts for the β-scission process and the pseudo first order rate constant for reaction with solvent (≤3 × 10^6^ s^–1^), and *k*_H_ is the second order rate constant for hydrogen abstraction from MPTP. Under the pseudo first order conditions of this experiment, [MPTP] > [^*t*^BuO^•^], a plot of *k*_obs_ vs the concentration of MPTP yields a straight line whose slope is *k*_H_.

**Figure 4:**
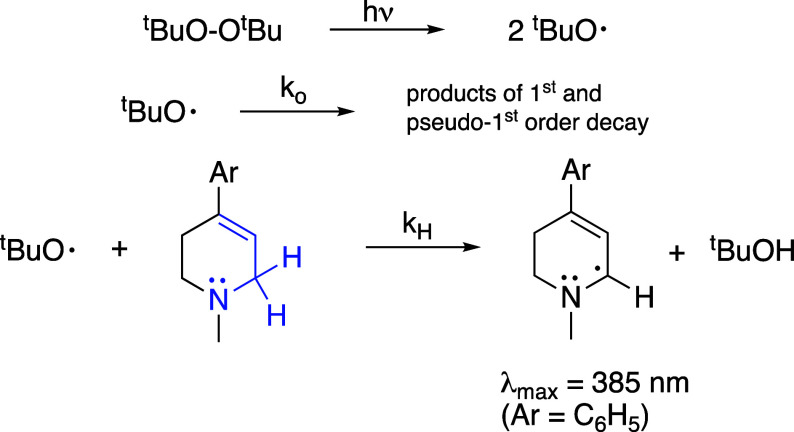
Generation of α-aminoalkyl radicals from tetrahydropyridines that are MPTP derivatives.

As Lalevée et al. have shown,^23^ at longer times (millisecond time regime) it is possible to measure the second order rate constant for reaction of aminoalkyl radicals with O_2_ by monitoring their decay under aerobic conditions. In the case of MPTP and derivatives, several of the dihydropyridinium species (DHP^+^) resulting from electron transfer have been previously characterized and shown to have unique UV signals. Accordingly, by this technique, if the reaction with oxygen generates a dihydropyridinium species either directly via electron transfer or through a peroxyl radical intermediate (Figure 5), it should be possible to detect the DHP^+^ product.

**Figure 5:**
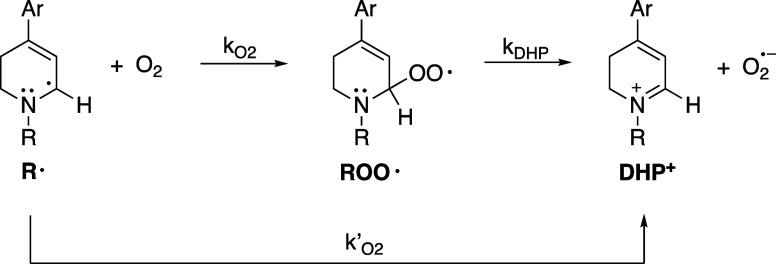
Reaction of α-aminoalkyl radicals (R^•^) with oxygen via (a) radical trapping to produce ROO^•^, possibly followed by decomposition to yield DHP^+^ and O_2_^•–^, or (b) electron transfer to form DHP^+^ and O_2_^•–^ directly.

Each of the MPTP derivatives examined gave rise to a transient signal in the region 360–410 nm via reaction with ^*t*^BuO^•^, which reached maximum intensity in approximately 500 ns. Figure 6 shows a representative spectrum and transient trace for MPTP derivative **2b** (*p*-ClC_6_H_6_); results for other derivatives are provided in the Supporting Information.

**Figure 6:**
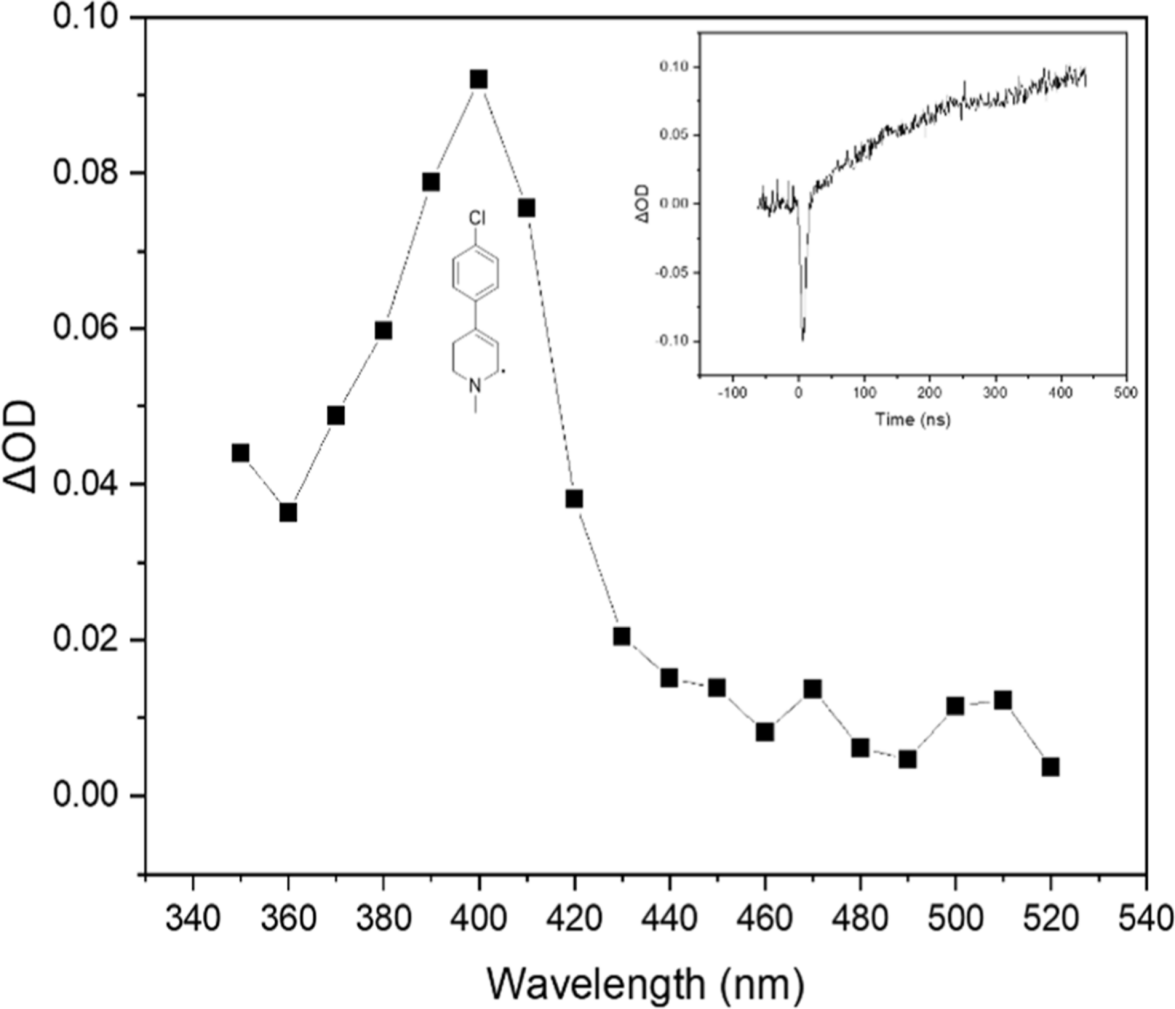
Transient absorption spectrum for the reaction of ^*t*^BuO^•^ with **1b** at 800 ns (anaerobic). (Insert: transient trace monitored at λ_max_ = 400 nm.)

Table 1 summarizes the pertinent spectroscopic data (theory and experiment) and calculated ionization potentials for the aminoalkyl radicals (R^•^) and dihydropyridiniums (DHP^+^) derived from **1** to **5**.

**Table 1:** Pertinent Spectroscopic Data for R^•^ and DHP^+^ Derived From Tetrahydropyridines **1**–**5** ^a^

derivative	R^•^ λ_max_ (exp)	R^•^ λ_max_ (*f*) (calc)	DHP^+^ λ_max_ (exp)	DHP^+^ λ_max_ (*f*) (calc)	IP (eV)
**MPTP**	385^b^	354 (0.203)	345^c^	377 (0.523)	
**1a**	380	366 (0.602)	340	370 (0.729)	3.70
**1b**	400	381 (0.690)	350	377 (0.815)	3.83
**1c**	400	383 (0.728)	350	385 (0.841)	3.85
**2**	360	343 (0.534)	420^d^	392 (0.748)	3.32
**3**	410	384 (0.594)		337 (0.488)	4.03
**4**	380	446 (0.626)	370	371 (0.842)	3.99
**5**	380	378 (0.654)		371 (0.768)	3.50

a R^•^ and DHP^+^ refer to aminoalkyl radicals and dihydropyridiniums, respectively, derived from **1** to **5**.

b Ref 25.

c Ref 27.

d Reported λ_max_ is 420 nm (ref 34).

As described above, rate constants for hydrogen atom abstraction were determined. The results are summarized in Table 2. For all of the compounds examined, *k*_H_ was on the order of 1.7–2.9 × 10^8^ M^–1^ s^–1^, virtually identical to that for MPTP. This result was not surprising, as the α-C–H bond in each of these compounds is, by far, the weakest bond all with a bond dissociation energy (BDE) of ca. 75 (±2) kcal/mol.^24^

**Table 2:** Pertinent Kinetic Data for the Reaction of ^*t*^BuO^•^ With Tetrahydropyridines **1**–**5**

derivative	*k*_H_ (10^8^ M^–1^ s^–1^)	*k*_O_2__ (10^8^ M^–1^ s^–1^)	*k*_DHP_ (10^8^ s^–1^)
**MPTP**	2.3^a^		
**1a**	1.7 (±0.4)	12	0.882
**1b**	2.2 (±0.5)	7	0.757
**1c**	1.9 (±0.4)	2	1.14
**2**	2.9 (±0.5)	9	11.3
**3**	2.5 (±0.3)		
**4**	2.0 (±0.3)	3	
**5**	2.6 (±0.3)	7	

a Ref 25.

There are two additional matters to consider: hydrogen abstraction may occur from other positions in the substrate, but is expected to be a minor contributor to the overall kinetics. The other C–H bonds in the tetrahydropyridine ring are much stronger with BDEs ≥87 kcal/mol,^24^ and it has already been shown that for MPTP, hydrogen atom abstraction from these positions by ^*t*^BuO^•^ occurs to an extent ≤25% or so.^25^ It is also critical to note that none of these radicals will have a UV absorption in the region studied because none have the extent of conjugation associated with MPTP^•^ (and related radicals).

Under aerobic conditions and at longer timeframes (ca. 20 μs), the radicals derived from *p*-substituted phenyl-substituted MPTP derivatives **1a**–**c** and **2** all decayed and a new transient species was observed with λ_max_ corresponding to that reported previously for the dihydropyridinium species (DHP^+^). These observations provide compelling evidence for the conversion R^•^ + O_2_ → DHP^+^ + O_2_^•–.^ Results for **2b** are shown in Figure 7, and provided for the other compounds in the Supporting Information. As seen in Figure 7, many of these traces seemed to exhibit an isosbestic point consistent with the proposed reaction, although this does not necessarily mean the conversion is direct (vide infra).

**Figure 7:**
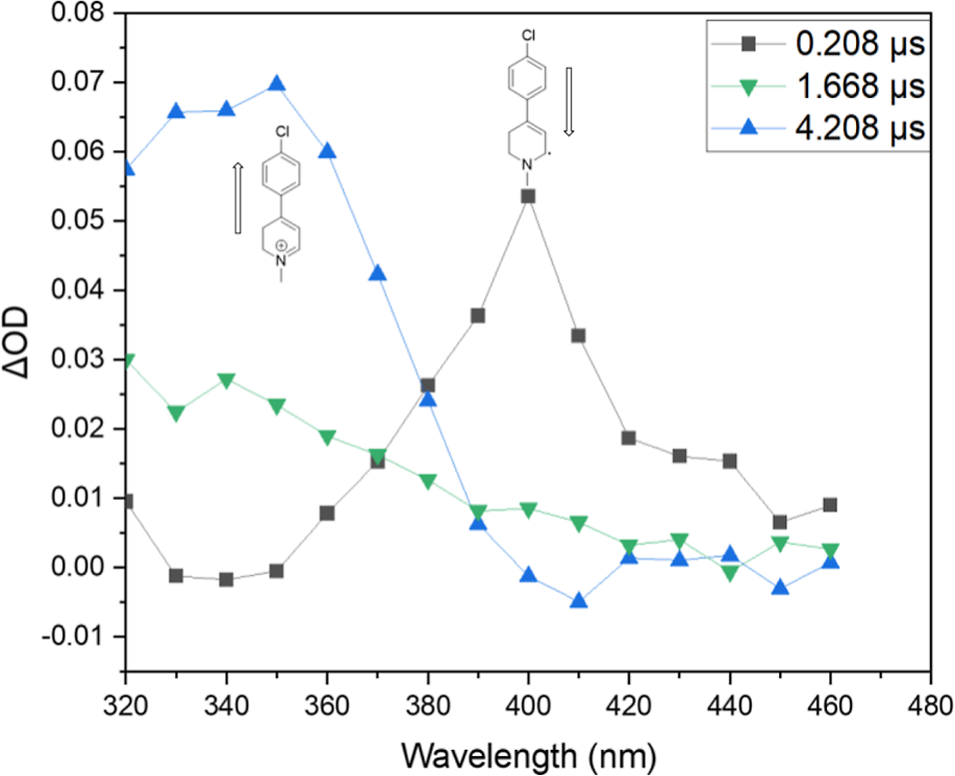
Transient absorption spectra for the reaction of ^*t*^BuO^•^ with tetrahydropyridine **1b** in the presence of O_2_ at various times. The peak assigned to R^•^ (400 nm) diminishes in intensity, and a new peak assigned to DHP^+^ appears (350 nm).

In the presence of O_2_, radicals derived from **3** and **5** underwent accelerated decay as expected in the presence of O_2_, but did not yield a new (observable) transient. This means either their reaction with O_2_ does not involve electron transfer to yield DHP^+^, or that DHP^**+**^ absorbs at the same wavelength as the radical itself. To probe some of these issues, molecular orbital calculations were performed to better understand the observed trends in the λ_max_ of R^•^ and DHP^+^ for the various MPTP derivatives. The calculations revealed that λ_max_ for both R^•^ and DHP^+^ were close to the experimental; λ_max_ for R^•^ tended to be underestimated by ca. 16 nm, while those for DHP^+^ were overestimated by ca. 30 nm. Perhaps more significantly, the calculations also suggest these two species might have nearly the same λ_max_ in some cases. For **3** and **5**, a unique peak attributable DHP^+^ was not observed experimentally. It is possible that λ_max_ for R^•^ and DHP^+^ derived from **3** and **5** were close, and that the electron transfer could not be detected. It may also be that these radicals do not undergo electron transfer with O_2_, but this seems unlikely. In general, α-aminoalkyl radicals are readily oxidized,^35^ and the α-C=C in the tetrahydropyridine derivatives further enhances their oxidizability.^24^ With the exception of **3** and **4**, the calculated ionization potentials for the aminoalkyl radicals derived from tetrahydropyridines are all very similar, so it seems unlikely that any of them are less susceptible to oxidation.

The calculations also revealed that for all the compounds studied, the oscillator strengths (*f*) for R^•^ and DHP^+^ are predicted to be similar (Table 1). In the context of Beer’s law, the predicted extinction coefficient (ε) is related to *f*.^36,37^ From this, it is reasonable to suppose that R^•^ and DHP^+^ might have similar extinction coefficients, and based upon the transient absorption spectra at various times in the presence of O_2_ (e.g., Figure 5; Figures S15–S18 in the Supporting Information), that a significant amount of R^•^ is converted to DHP^+^ and that this reaction is not a minor pathway for radical decay.

However, in all cases, the decay of R^•^ occurs 2–3 times faster than DHP^+^ formation, suggesting the electron transfer may not be concerted and that an intermediate is formed (illustrated in Figure 8 for **1b**, and provided in the Supporting Information for the other systems). As noted earlier, the reaction of a carbon-centered radical with O_2_ generally leads to a peroxyl radical, and we propose that a short-lived peroxyl radical intermediate is involved in this reaction as well (Figure 5). Fitting of the transient traces for DHP^+^ appearance led to the derived values of *k*_DHP_; the half-life (*t*_1/2_) for the peroxyl radical intermediates were on the order of a nanosecond. Thus, the overall reaction is effectively an inner-sphere electron transfer process proceeding through a peroxyl radical intermediate. Like the precursor radical, DHP^+^ is highly conjugated which likely provides the driving force for the overall electron transfer process.

**Figure 8:**
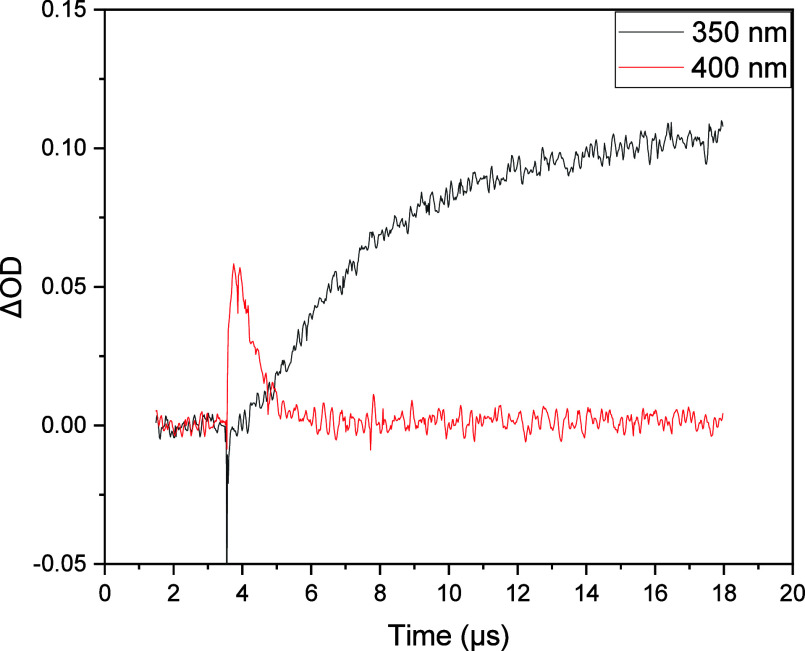
Transient traces for the disappearance of R^•^ derived from **1b** (400 nm) and appearance of DHP^+^ (350 nm). The results suggest that the transformation R^•^ → DHP^+^ is not concerted and likely involves a peroxyl radical intermediate.

## Conclusion

Tetrahydropyridines such as MPTP and derivatives are unique in that they possess an exceptionally weak C–H bond that is α- both to a nitrogen and a C=C. The combined effect of these radical stabilizing substituents is to lower the BDE to ca. 75 kcal/mol (vs ≥90 kcal/mol for an ordinary tertiary amine). Moreover, like other aminoalkyl radicals, the radicals derived from hydrogen atom abstraction at this position react with oxygen at a nearly diffusion-controlled rate, and are readily oxidized. The initial product of this reaction is most likely a peroxyl radical, which in the cases described herein, further decays to form DHP^+^ and O_2_^•–^. These results further demonstrate the unique chemistry associated with tetrahydropyridines such as MPTP, and possibly why in the context of MAO-catalyzed oxidations, these compounds are good substrates and/or inhibitors. Further, in addition to regenerating the flavin moiety in MAO to complete the catalytic cycle, O_2_ may also be directly involved in oxidizing MPTP^•^ to MPDP^+^.

## Supplementary Material



## Notes

Included are transient absorption spectra for reaction of *t*-butoxyl radical with compounds **1**–**5**, plots of *k*_obs_ vs substrate concentration to determine *k*_H_, transient absorption spectra and traces for reaction of *t*-butoxyl radical with **1**–**5** in the presence of O_2_ (PDF)
