# Supplementary material for: Reaction of O2 with α‑Aminoalkyl Radicals Derived from Tetrahydropyridines
Source: J Phys Chem A. 2025 Jun 11;129(24):5337–42. doi: 10.1021/acs.jpca.5c02967 (PMC12186616; doi:10.1021/acs.jpca.5c02967)
Supplement: Supplementary file 1 [file jp5c02967_si_001.pdf]

Supporting Information for

Reaction of O_2 with α-Aminoalkyl Radicals Derived from Tetrahydropyridines

Paul Venturo, Neal Castagnoli, Jr., and James M. Tanko
Department of Chemistry
Virginia Polytechnic Institute and State University
Blacksburg, VA 24061

Contents

Transient absorption spectrum for the reaction of $^tBuO•$ with compounds **1 - 5** S2

Plots of k_{obs} vs. substrate concentration to determine k_H S6

Transient absorption spectra for the reaction of $^tBuO•$ with tetrahydropyridines **1 - 5** in the presence of O_2 at various times. S10

Transient traces for the disappearance of **R•** derived from tetrahydropyridines **1 - 5** in the presence of O_2, and in some instances, the appearance of DHP^+ S14

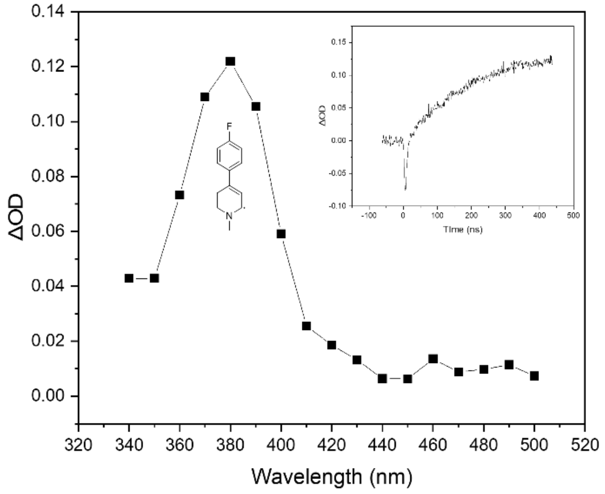

Figure S1. Transient absorption spectrum for the reaction of ^{t}BuO• with **1a** at 400 ns. (Insert: Transient trace monitored at 380 nm.)

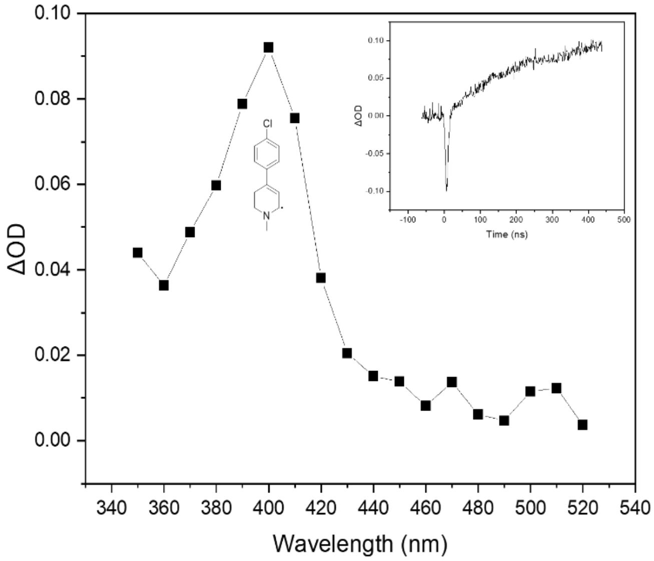

Figure S2. Transient absorption spectrum for the reaction of ^{t}BuO• with **1b** at 400 ns. (Insert: Transient trace monitored at 400 nm.)

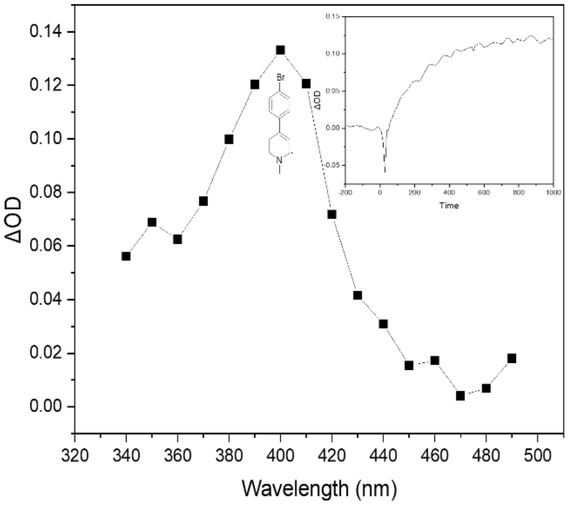

Figure S3. Transient absorption spectrum for the reaction of ${}^{t}BuO\bullet$ with **1c** at 1000 ns. (Insert: Transient trace monitored at 400 nm.)

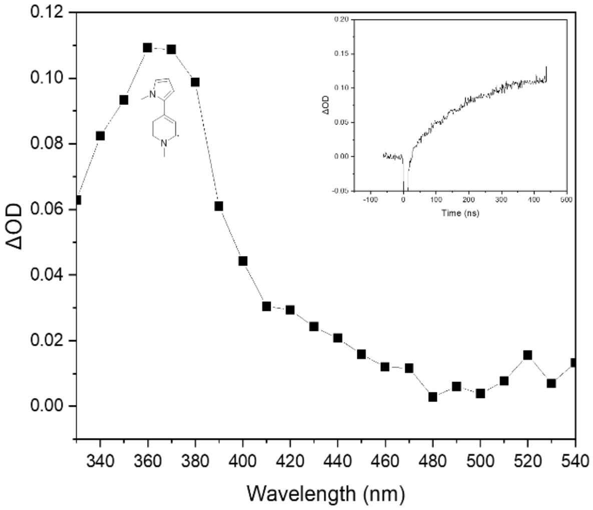

Figure S4. Transient absorption spectrum for the reaction of ${}^{t}BuO\bullet$ with **2** at 400 ns. (Insert: Transient trace monitored at 360 nm.)

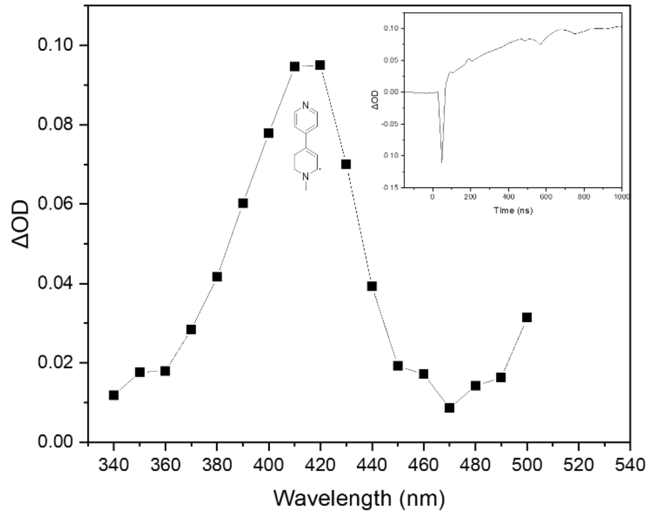

Figure S5. Transient absorption spectrum for the reaction of ^{t}BuO• with **3** at 1000 ns. (Insert: Transient trace monitored at 410 nm.)

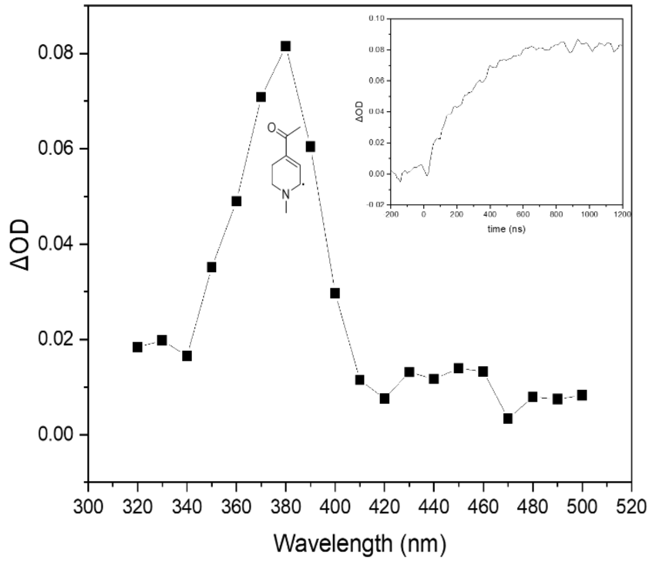

Figure S6. Transient absorption spectrum for the reaction of ^{t}BuO• with **4** at 1200 ns. (Insert: Transient trace monitored at 380 nm.)

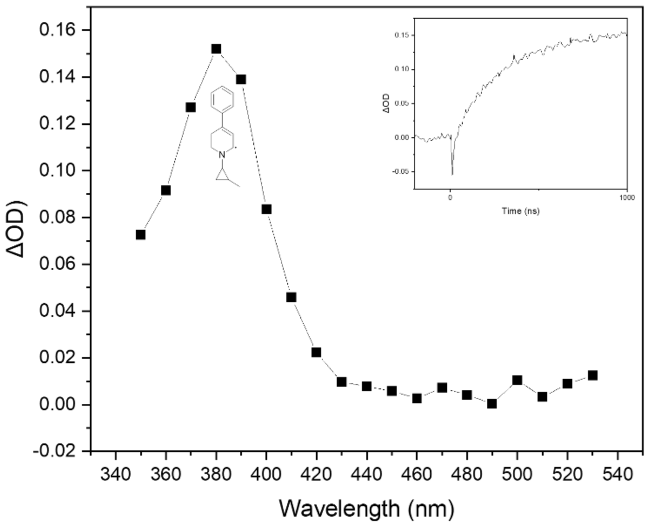

Figure S7. Transient absorption spectrum for the reaction of ^{t}BuO• with **5** at 1000 ns. (Insert: Transient trace monitored at 380 nm.)

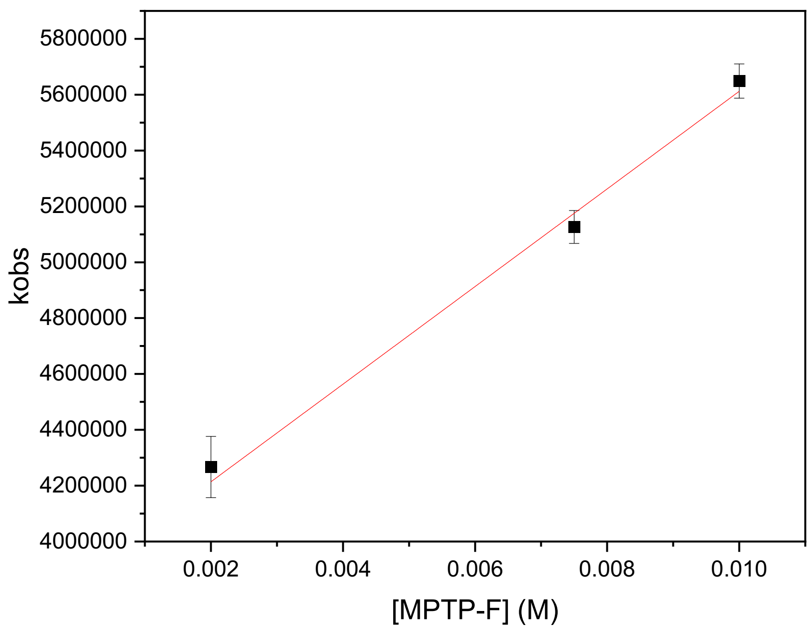

Figure S8. Plot of k_{obs} vs. [**1a**] to determine k_H. Slope and standard error: 1.70048E+08 ± 3.8562E+07

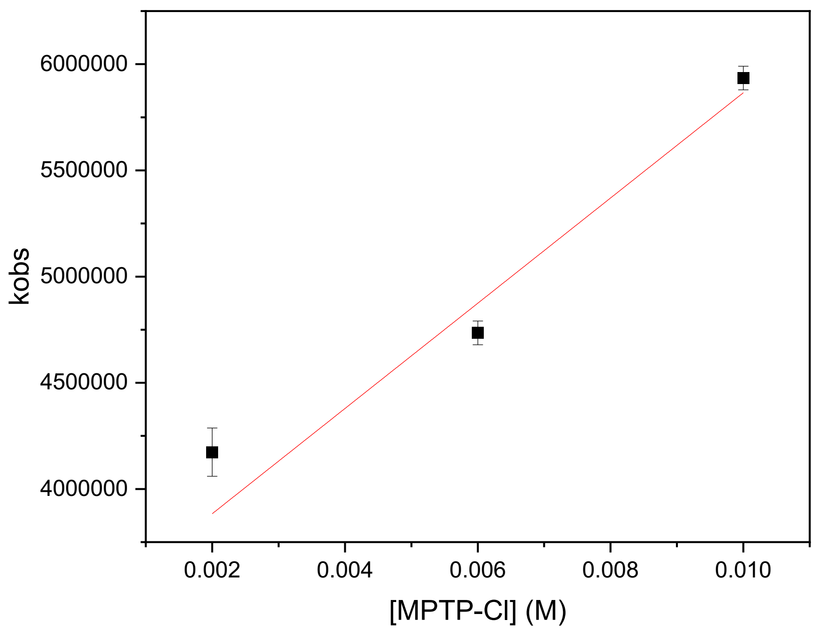

Figure S9. Plot of k_{obs} vs. [**1b**] to determine k_H. Slope and standard error: 2.2014E+08 ± 4.6026E+07

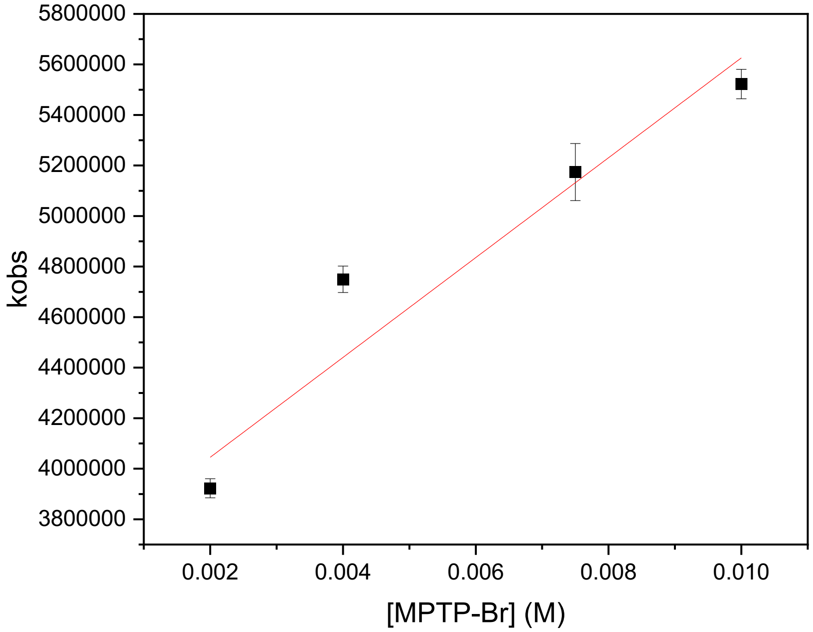

Figure S10. Plot of k_{obs} vs. [**1c**] to determine k_H. Slope and standard error: 1.8543E+08 ± 3.8562E+07

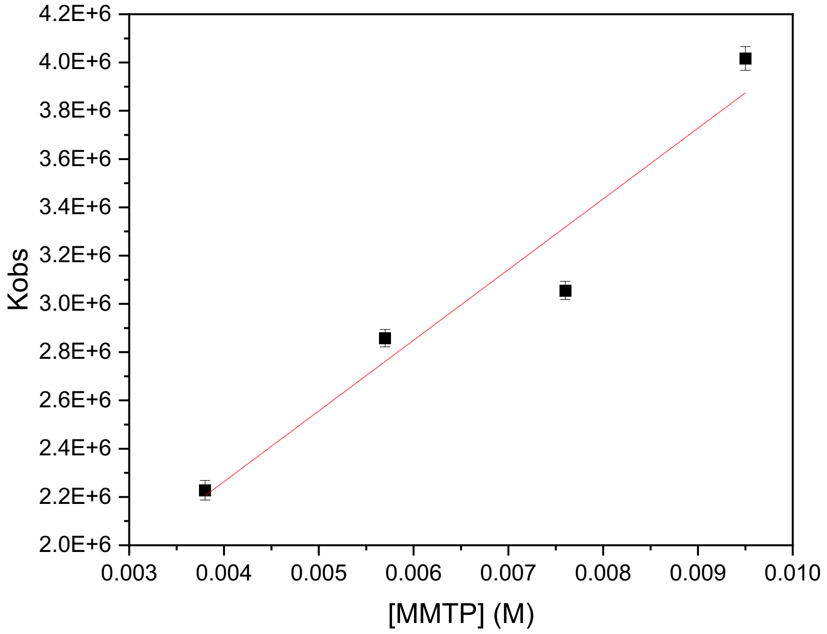

Figure S11. Plot of k_{obs} vs. [**2**] to determine k_H. Slope and standard error: 2.9285E+08 ± 5.2357E+07

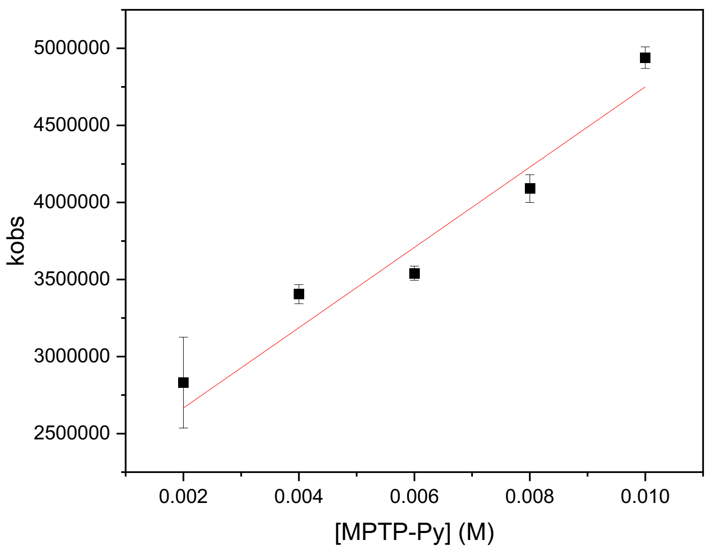

Figure S12. Plot of k_{obs} vs. [**3**] to determine k_H. Slope and standard error: 2.4514E+08 ± 3.3496E+07

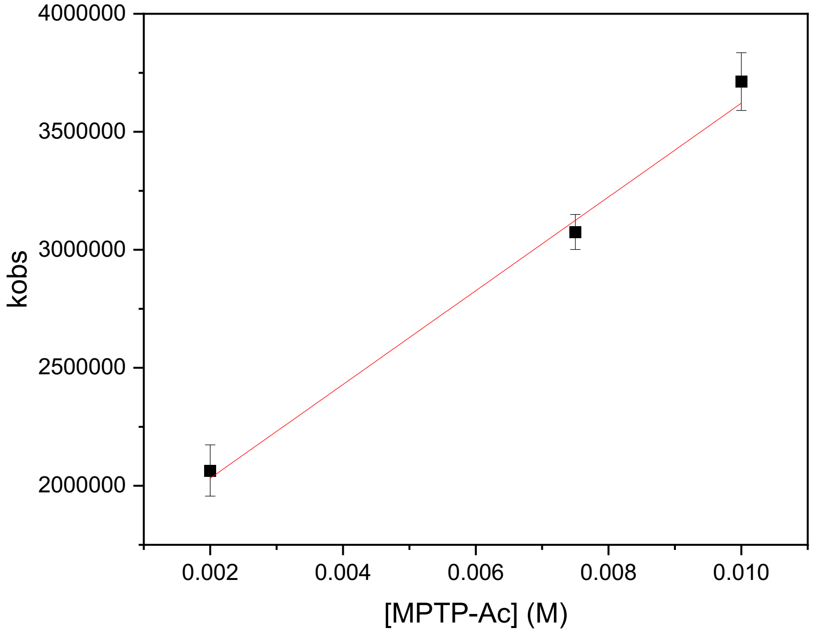

Figure S13. Plot of k_{obs} vs. [**4**] to determine kH. Slope and standard error: 2.02343E+08 ± 1.68372E+07

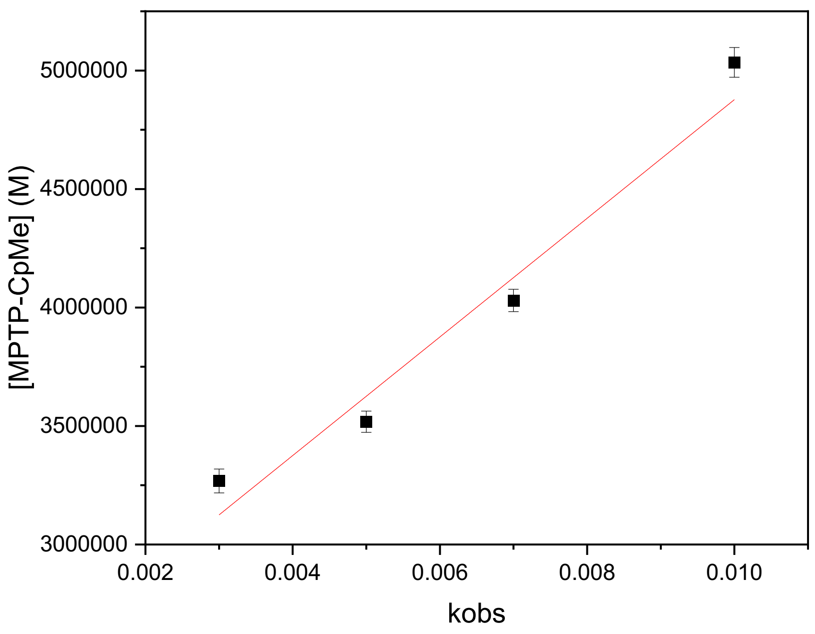

Figure S14. Plot of k_{obs} vs. [**5**] to determine k_H. Slope and standard error: 2.57266E+08 ± 3.41744E+07

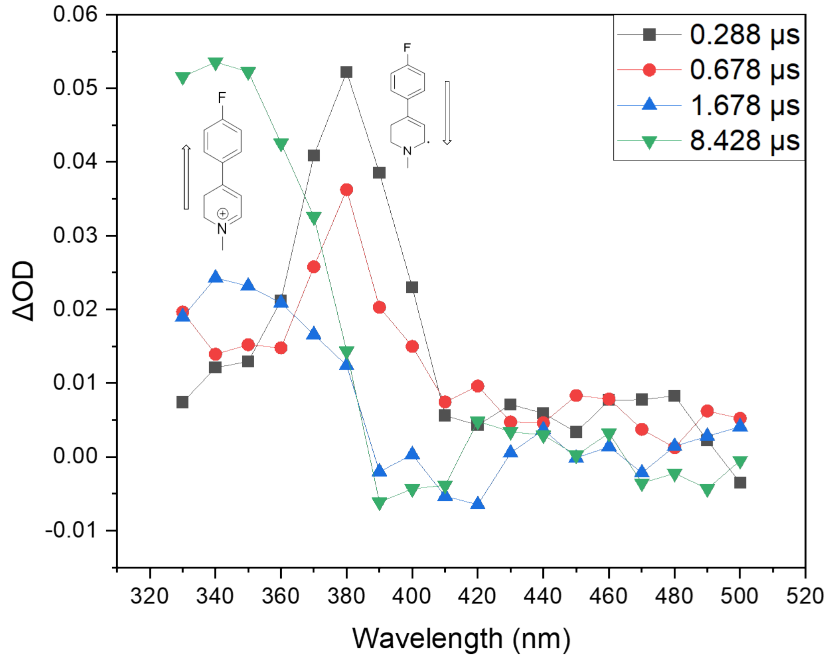

Figure S15. Transient absorption spectra for the reaction of ${}^{t}BuO\bullet$ with tetrahydropyridine **1a** in the presence of O_2 at various times.

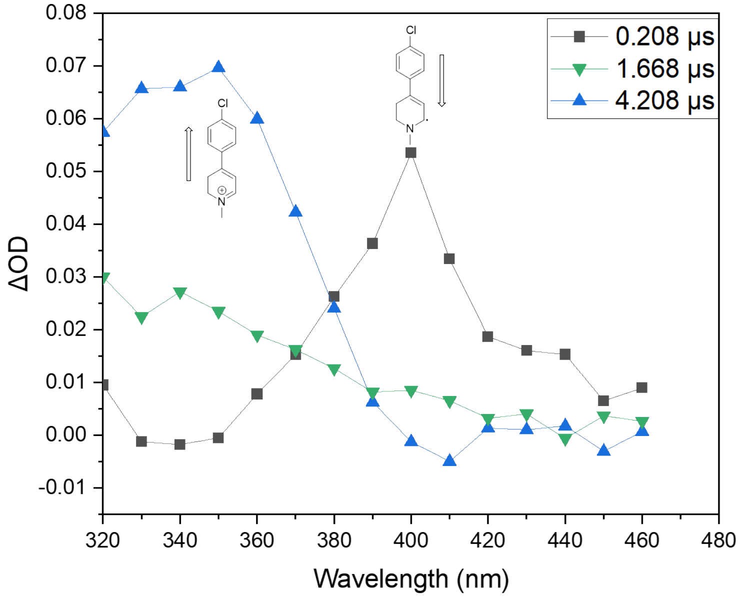

Figure S16. Transient absorption spectra for the reaction of ${}^{t}BuO\bullet$ with tetrahydropyridine **1b** in the presence of O_2 at various times.

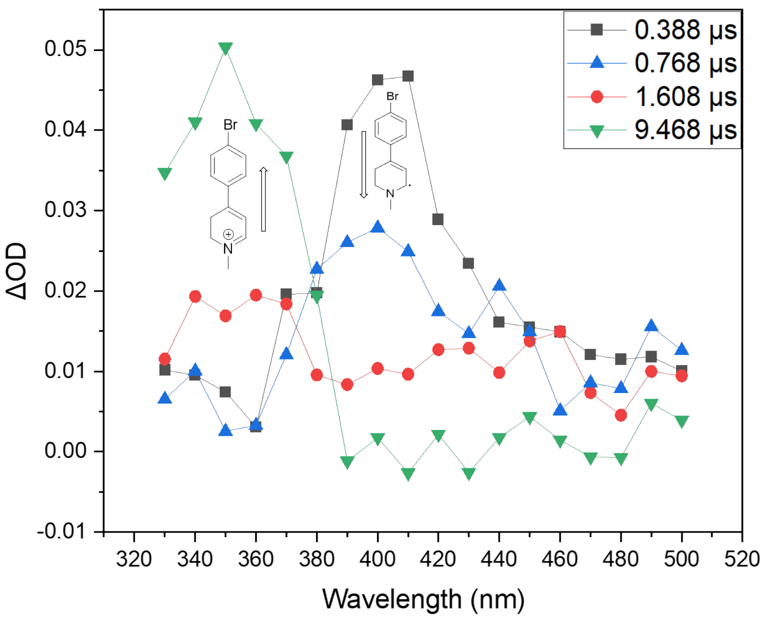

Figure S17. Transient absorption spectra for the reaction of ^{t}BuO• with tetrahydropyridine **1c** in the presence of O_2 at various times.

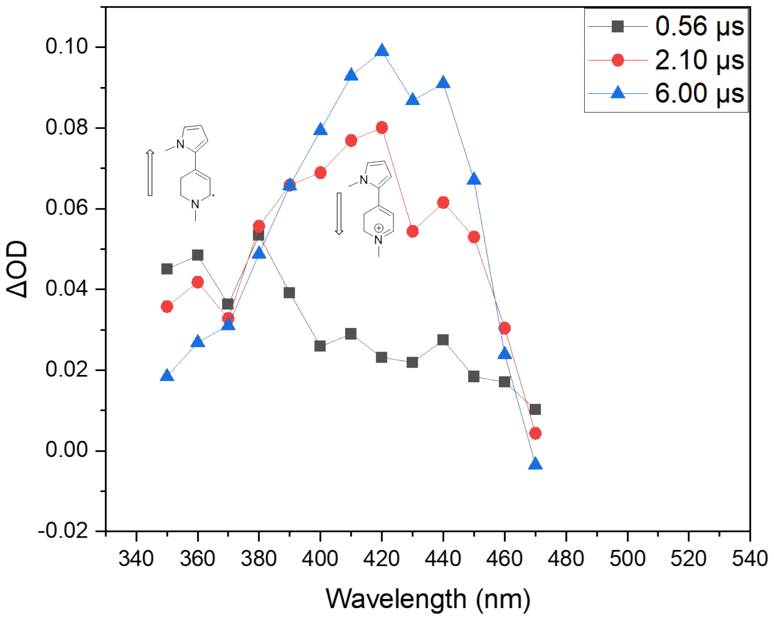

Figure S18. Transient absorption spectra for the reaction of ^{t}BuO• with tetrahydropyridine **2** in the presence of O_2 at various times.

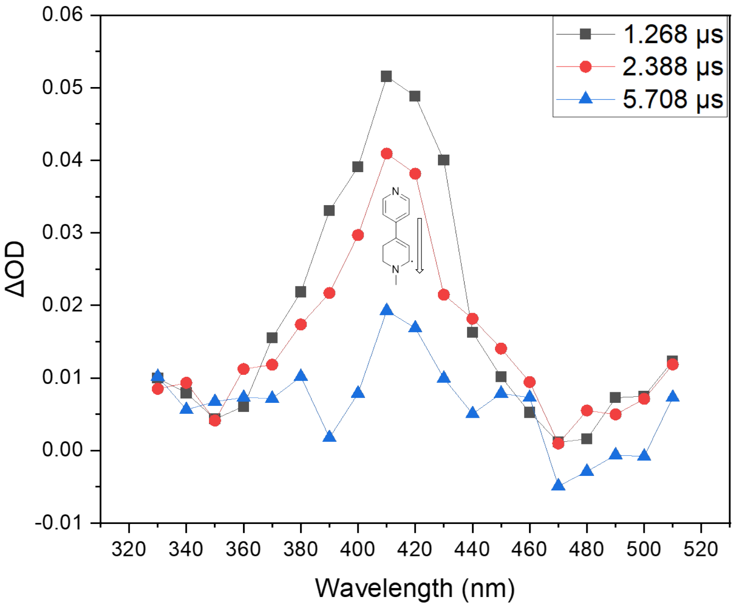

Figure S19. Transient absorption spectra for the reaction of ^{t}BuO• with tetrahydropyridine **3** in the presence of O_2 at various times. No new peaks are observed as the R• signal decays.

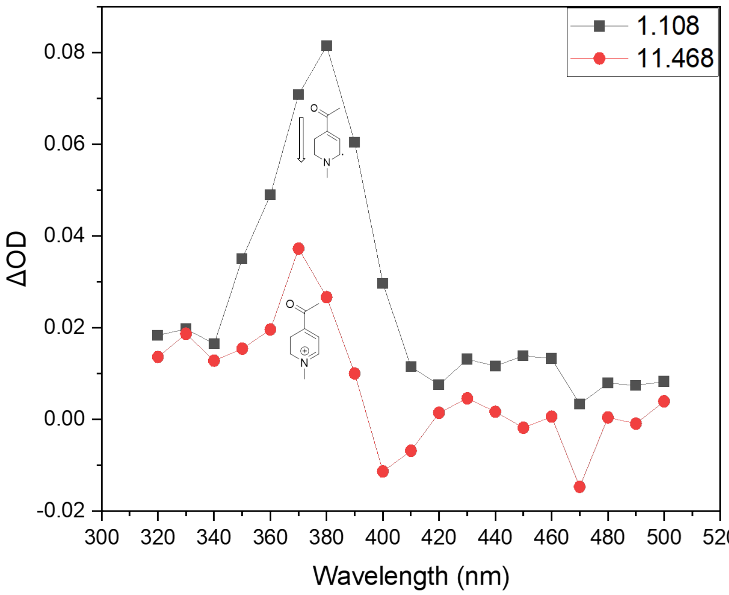

Figure S20. Transient absorption spectra for the reaction of ^{t}BuO• with tetrahydropyridine **4** in the presence of O_2 at various times.

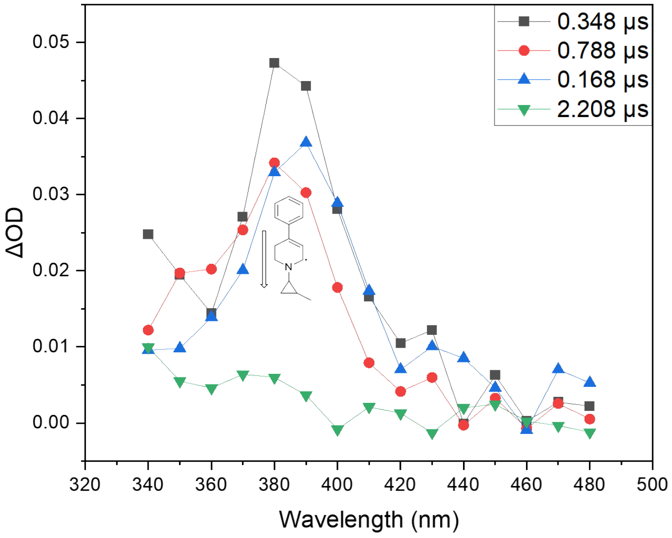

Figure S21. Transient absorption spectra for the reaction of ^{t}BuO• with tetrahydropyridine **5** in the presence of O_2 at various times. No new peaks are observed as the R• signal decays.

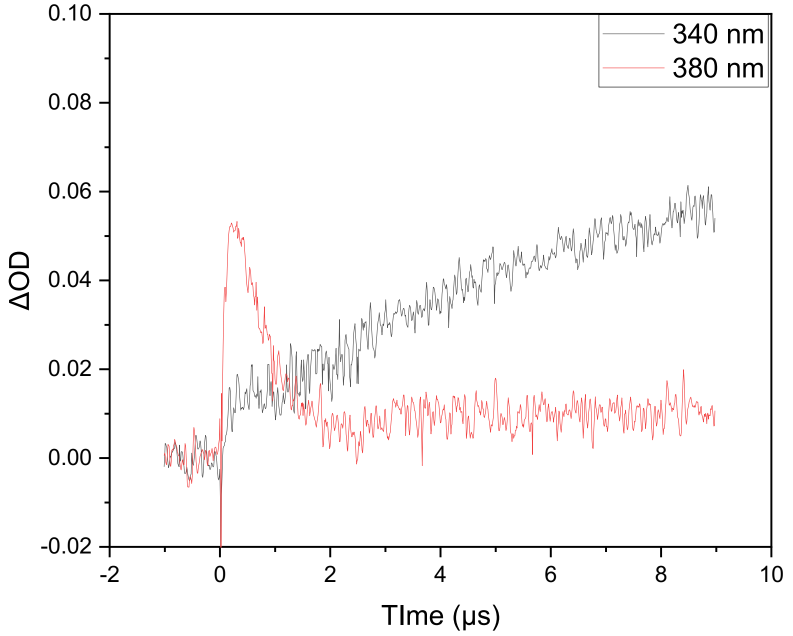

Figure S22. Transient traces for the disappearance of **R•** derived from **1a** (340 nm) and appearance of **DHP⁺** (380 nm) in the presence of O_2.

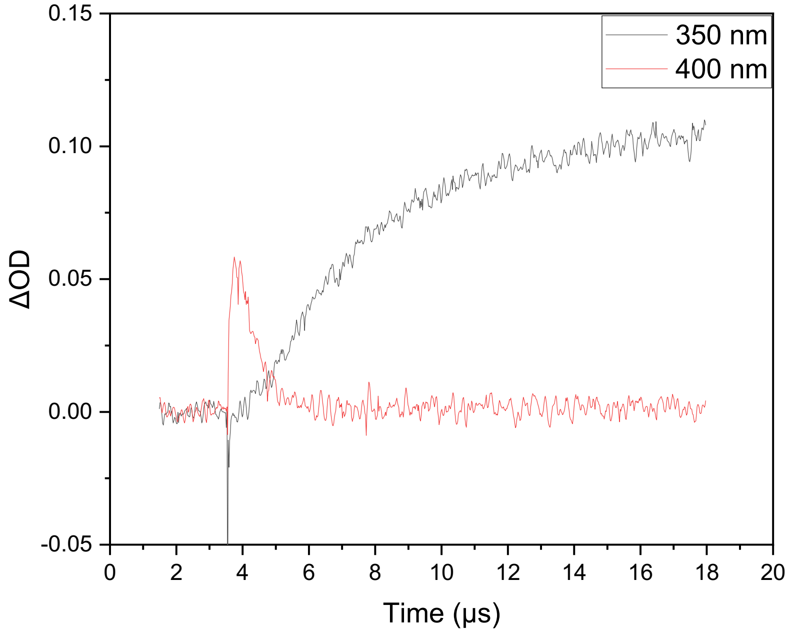

Figure S23. Transient traces for the disappearance of **R•** derived from **1b** (400 nm) and appearance of **DHP⁺** (350 nm) in the presence of O_2.

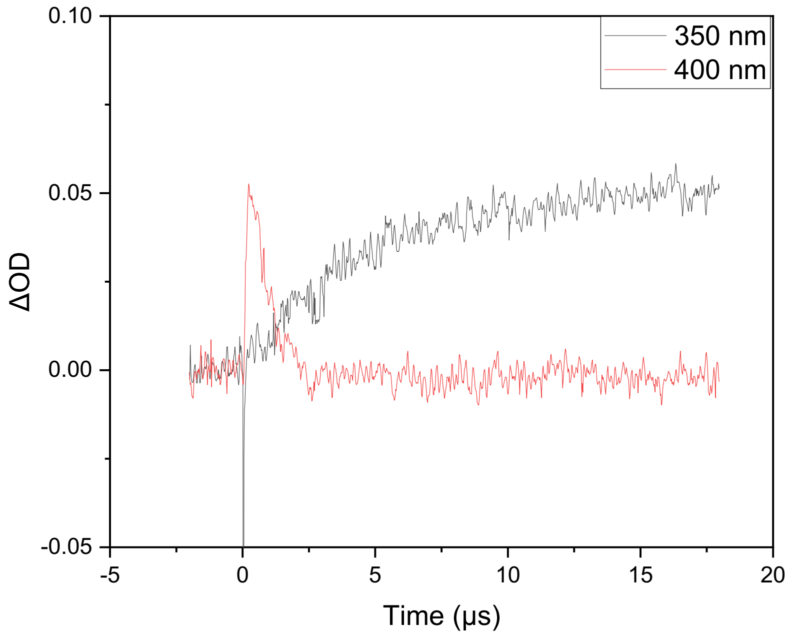

Figure S24. Transient traces for the disappearance of **R•** derived from **1c** (400 nm) and appearance of **DHP$^+$** (350 nm) in the presence of O_2.

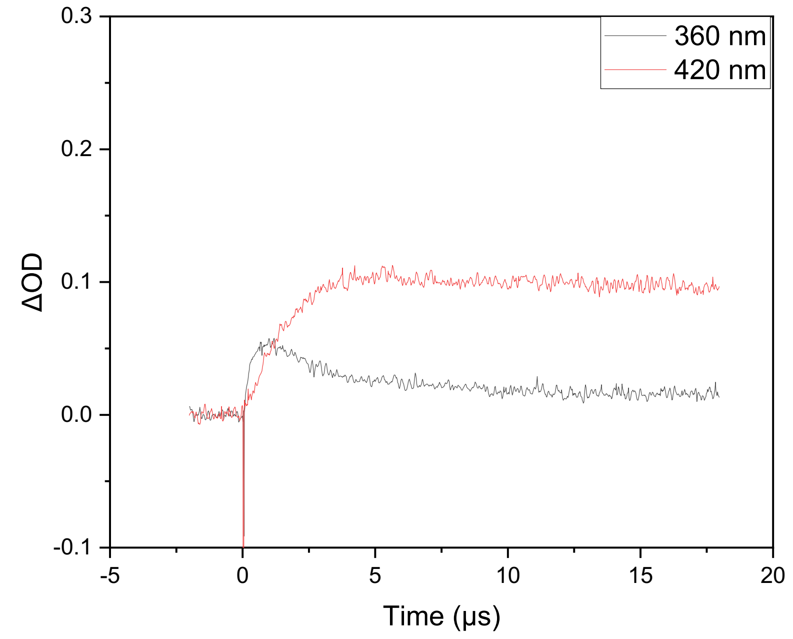

Figure S25. Transient traces for the disappearance of **R•** derived from **2** (360 nm) and appearance of **DHP$^+$** (420 nm) in the presence of O_2.

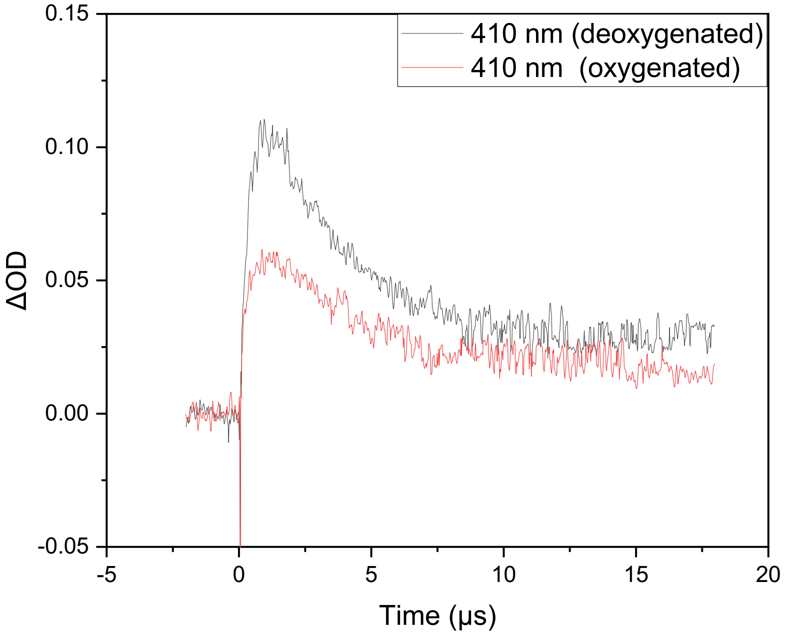

Figure S26. Transient traces for the decay of **R•** derived from **3** in the presence and absence of O_2.

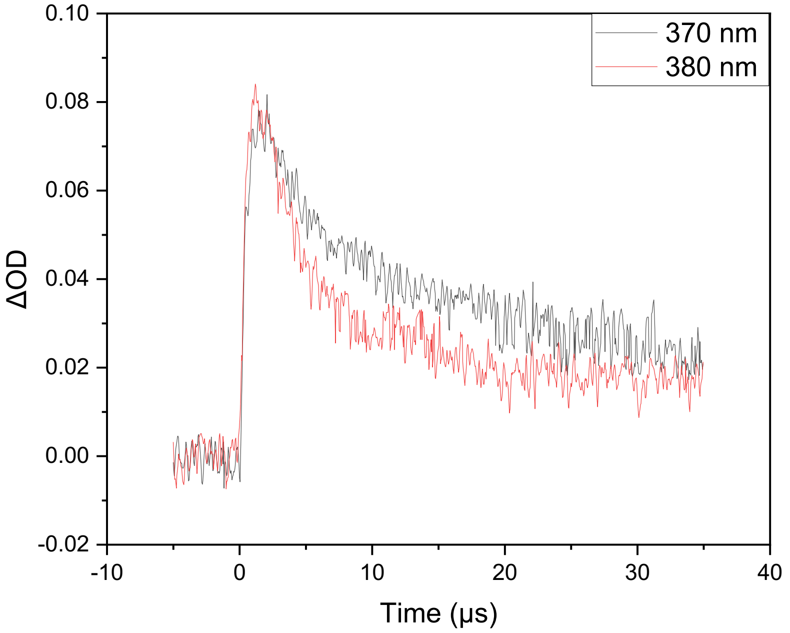

Figure S27. Transient trace for the disappearance of **R•** derived from **4** (380 nm) in the presence of O_2. Also shown is the transient trace monitored at 370 nm.

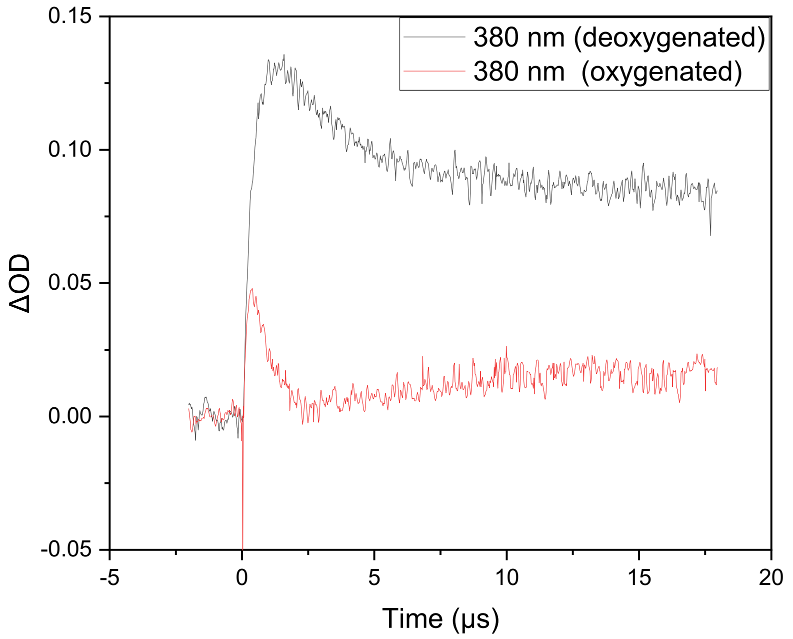

Figure S28. Transient traces for the disappearance of **R•** derived from **5** (380 nm) in the presence and absence of O_2.

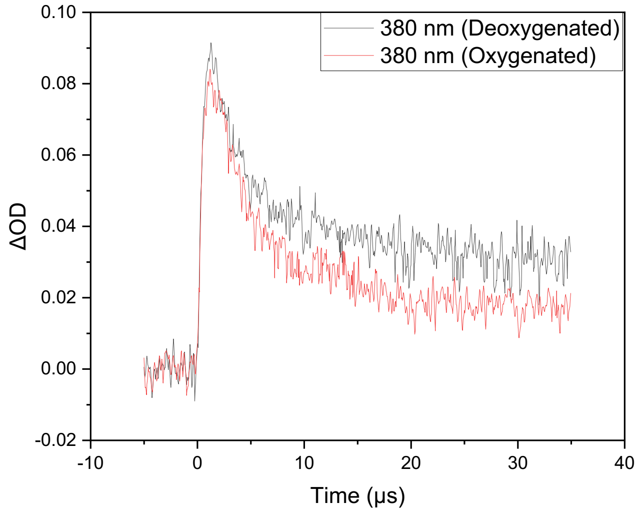

Figure S29. Transient traces for the disappearance of **R•** derived from **4** (380 nm) in the presence and absence of O_2.
